# Effects of catheter‐based renal denervation on renin‐aldosterone system, catecholamines, and electrolytes: A systematic review and meta‐analysis

**DOI:** 10.1111/jch.14590

**Published:** 2022-11-02

**Authors:** Xiangyu Yang, Lede Lin, Zhipeng Zhang, Xiaoping Chen

**Affiliations:** ^1^ Department of Cardiology, West China Hospital Sichuan University Chengdu China; ^2^ Department of Urology, West China Hospital Sichuan University Chengdu China

**Keywords:** catecholamines, electrolytes, Raas, renal denervation, sympathetic nervous system

## Abstract

In recent years, catheter‐based renal denervation (RDN) has emerged as a promising instrumental therapy for hypertension. The interruption of sympathetic nervous system was regarded as a possible mechanism for RDN regulating blood pressure. While the results reflected by renin‐angiotensin‐aldosterone system (RAAS), catecholamines and electrolytes remained inconsistent and was never systematically assessed. Pubmed, Embase, and Web of Science were comprehensively searched from inception to September 5, 2021. Studies that evaluated the effects of RDN on RAAS, catecholamines, and electrolytes were identified. Primary outcomes were changes in RAAS hormones after RDN, and secondary outcomes involved changes in plasma norepinephrine, serum, and urinary sodium and potassium. Out of 6391 retrieved studies, 20 studies (two randomized controlled studies and 18 observational studies) involving 771 persons were eventually included. Plasma renin activity had a statistically significant reduction after RDN (0.24 ng/mL/h, 95% CI 0.04 to 0.44, *P* = .02). While no significant change was found regarding plasma aldosterone (1.53 ng/dL, 95% CI ‐0.61 to 3.67, *P* = .16), norepinephrine (0.42 nmol/L, 95% ‐0.51 to 1.35, *P* = 0.38), serum sodium and potassium (0.16 mmol/L, 95% CI ‐0.17 to 0.49, *P* = .34; ‐0.02 mmol/L, 95% CI ‐0.09 to 0.04, *P* = .48, respectively), and urinary sodium and potassium (3.95 mmol/24 h, 95% CI ‐29.36 to 37.26, *P* = .82; 10.22 mmol/24 h, 95% CI ‐12.11 to 32.54, *P* = .37, respectively). In conclusion, plasma renin activity significantly decreased after RDN, while no significant change was observed in plasma aldosterone, plasma norepinephrine, and serum and urinary electrolytes.

## 1 INTRODUCTION

Hypertension (HTN) remains one of the heaviest burdens of public health worldwide, as the number of adults affected by HTN maintains an upward trend, which is predicted to reach a total of 1.56 billion in 2025.^1^ Though with the popularization of antihypertensive drugs, the treatment and control rates of HTN in some countries were still low.^2^ According to existed reports, blood pressure (BP) was still not controlled in 13.72‐16.24% hypertensives under treatment of at least triple combinations of drugs,^3^ which was defined as resistant hypertension (RH). To meet the growing needs and achieve better BP control, catheter‐based renal denervation (RDN) emerged as a promising interventional approach to complement pharmaceutical therapies in HTN patients, especially in those with RH.^4^, ^5^, ^6^

The important role of the sympathetic nervous system in the pathogenesis of HTN has been proved by previous research.^7^ The blockage of renal efferent sympathetic nerves was also regarded as part of the mechanisms of RDN reducing BP, through altering renal blood flow, increasing urinary salt excretion, and decreasing renin release from the kidney.^8^ While the results regarding the direct effect of RDN on the sympathetic nervous system, reflected by changes in plasma renin, aldosterone, catecholamines, and subsequent serum and urinary electrolytes, are inconsistent and have never been systematically assessed. The objective of this study was to undertake a systematic review and meta‐analysis of research to determine whether such changes existed after the procedure of catheter‐based RDN in HTN patients.

## 2 METHODS

We conducted the systematic review and meta‐analysis according to the PRISMA (Preferred Reporting Items for Systematic reviews and Meta‐analyses) guidelines.^9^

### 2.1 Search strategy

The goal of the search was to find studies that documented the changes in plasma renin, plasma aldosterone, plasma and urinary catecholamines, and serum and urinary electrolytes in hypertensive objectives after RDN. The following databases were searched from their inception until September 5, 2021, including Embase, Pubmed, and Web of Science. Each query group was created using Mesh‐terms along with free terms (renal denervation, renal‐angiotensin‐aldosterone system, catecholamines, sodium, potassium), and was eventually combined into a single search. No restriction was placed on the publication date, sample size, follow‐up time, or language type. Detailed search strategies are available in Supplementary file 1.

### 2.2 Study selection

The search results were screened independently by two reviewers. Inclusion criteria were studies reporting data on plasma renin, plasma aldosterone, plasma and urinary catecholamines, serum and urinary sodium or potassium before and after RDN procedure in HTN patients. Studies with at least one outcome of interest above were included. Only full‐text published articles were qualified for inclusion. Exclusion criteria were as follows: 1. Conference abstracts, case reports, reviews, editorials and letters; 2. Animal studies; 3. Articles with incomplete data; 4. Studies not targeted at hypertension patients. As for publications from the same population, reporting on different follow‐up period data, only the one with the longest follow‐up time was used for the overall analysis. Discrepancies in the screening results between the two reviewers were solved by further discussion and consensus.

### 2.3 Outcomes

The primary outcomes were changes in RAAS hormones after RDN, including plasma renin and plasma aldosterone. The secondary outcomes involved changes in plasma catecholamines, serum and urinary sodium and potassium, and blood pressure.

### 2.4 Data extraction and quality assessment

Two reviewers independently extracted data and assessed study quality. Information about studies characteristics (first author, year of publication, country, study design, sample size, type of patients, follow‐up time, type of RDN catheter), patients characteristics (mean age), and outcomes data (changes in plasma renin, plasma aldosterone, plasma catecholamines, plasma and urinary sodium and potassium, blood pressure) was extracted. With regard to studies reporting outcome data with inconsistent units, or those presenting data not in the form of mean ± standard deviation, we tried to convert them as possible using reasonable methods,^10^, ^11^, ^12^ before performing analysis (ng/mL/h for renin; ng/dL for aldosterone; nmol/L for norepinephrine; mmol/L for serum electrolytes; mmol/24 h for urinary electrolytes; mmHg for blood pressure). Only data from the RDN treatment arm was extracted for analysis.

Quality assessment was conducted on all included articles. For all the studies, methodological index for non‐randomized studies (MINORS)^13^ was applied in the assessment. MINORS comprised 12 items in total, including eight items for non‐comparative studies, and four additional items for comparative items. Each item should be scored from 0 to 2, representing not reported, reported but inadequate, and reported and adequate, respectively. The global ideal score was 16 for single‐arm studies. A total score of more than 12 points indicated high quality, 8–12 points indicated intermediate quality, and a score of less than 8 points indicated low quality.

### 2.5 Data synthesis and analysis

All statistical analyses were performed using Review Manager 5.3 software (Cochrane, London, UK). Pooled outcomes were reported as mean difference (MD) with 95% confidence interval (CI), in a random model. I^2^ test was utilized to detect potential heterogeneity. I^2^ ≤ 50% was considered to have acceptable heterogeneity. Meta‐regression analysis was used to evaluate the relationship between plasma renin activity (PRA) and BP. Statistical power was set at 0.05 on two sides. The results were presented as forest plots. Funnel plots were applied to detect potential publication bias. Egger test and Begg test were used to calculate significance of publication bias. If Egger test or Begg test was significant, then trim‐and‐fill analysis was utilized to input linear estimator to adjust publication bias.

## 3 RESULTS

### 3.1 Study selection and characteristics

**FIGURE 1:**
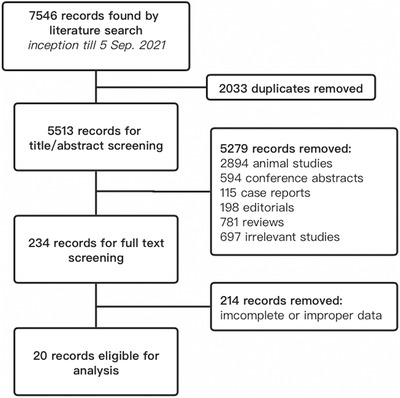
Flowchart of study selection

The search strategy identified a total of 7546 records from the three databases. After removing the duplicates and excluding results not meeting the inclusion and exclusion criteria, 20 articles were eventually retrieved for data extraction. Detailed process is presented in Figure 1.

The studies were published between 2011 and 2021. Among the 20 identified articles, 2 of them were RCTs, and the other 18 were observational studies. Most of the included studies reported on patients from Europe, Australia, and East Asia. A total of 771 persons were involved. The mean age of the patients from each study ranged from 51.2 to 68 years. Follow‐up duration ranged from 0.5 to 72 months. Ten studies reported data on plasma renin,^14^, ^15^, ^16^, ^17^, ^18^, ^19^, ^20^, ^21^, ^22^, ^23^ nine studies on plasma aldosterone,^14^, ^15^, ^16^, ^18^, ^19^, ^21^, ^22^, ^23^, ^24^ two studies on plasma norepinephrine,^20^, ^25^ seven studies on serum sodium,^23^, ^24^, ^26^, ^27^, ^28^, ^29^, ^30^ nine studies on serum potassium,^23^, ^24^, ^26^, ^27^, ^28^, ^29^, ^30^, ^31^, ^32^ five studies on urinary sodium,^18^, ^20^, ^24^, ^26^, ^33^ and two studies on urinary potassium.^24^, ^26^ The characteristics of included studies are summarized in Table 1.

**TABLE 1:** Baseline characteristics of included studies

First Author	Year	Area	Study Design	Sample Size	Age, y	Follow‐up, m	Catheter Type	Outcomes
M Voskuil	2011	Netherlands	Observational Study	11	68 ± 12	1	UK	RAAS
H Ahmed	2012	Czech Republic	Observational Study	5	–	3	Irrigated‐tip Celsius Thermocool ablation catheter	RAAS
W Li	2013	China	Observational Study	10	52.2 ± 11.9	0.5	Celsius	RAAS
M Hamza	2014	Egypt	Observational Study	55	58 ± 7	6	Symplicity	RAAS
J Poss	2015	Germany	Observational Study	137	63 ± 1	6	Symplicity	Electrolytes RAAS
S Ewen	2015	Italy	Observational Study	30	61.9 ± 9.9	6	Symplicity Flex	RAAS
LC Dobrowolski	2018	Italy	Observational Study	21	60(53‐70)	1.5	Symplicity	Electrolytes RAAS Catecholamines
L Feyz	2020	Netherland	Observational Study	60	64 ± 9	6	Paradiese, Vessix V2™, Symplicity, OneShot, EnligHTN	RAAS Catecholamines
CJ Kim	2021	South Korea	Observational Study	16	56.5 ± 14.8	3	DENEX	RAAS
F Mahfoud	2021	Eight countries	RCT	126	–	3	Symplicity Spyral, Symplicity G3	Electrolytes RAAS
C Ott	2017	Germany	Observational Study	41	61.0 ± 9.2	6	Symplicity and Flex	Electrolytes RAAS
M Ezzahti	2014	Netherlands	Observational Study	17	51.2 ± 9.4	6	Symplicity	Catecholamines
D Hering	2012	Australia	Observational Study	15	61 ± 9	3,6	UK	Electrolytes
D Hering	2013	Poland	Observational Study	40	60 ± 11	3	Symplicity	Electrolytes
D Hering	2016	Australia	Observational Study	65(G1), 16(G2), 10(G3)	63 ± 10(G1) 63 ± 11(G2) 67 ± 8(G3)	3,6	Symplicity	Electrolytes
J Rosa	2017	Czech Republic	RCT	52	56 ± 12	24	Symplicity	Electrolytes
U Kampmann	2017	Denmark	Observational Study	8	62.5 ± 2.55	6	Simplicity	Electrolytes
C Tsioufis	2014	Greece	Observational Study	14	55.4 ± 8.9	6	EnligHTN	Electrolytes
OU Bergland	2020	Norway	RCT	9	57 ± 10.9	6,36,72	UK	Electrolytes
Y Vuignier	2018	Switzerland	Observational Study	13	56.1 ± 9.9	1,12	Symplicity Flex, EnligHTN IV	Electrolytes

RCT, randomized control trials; RAAS, renin‐angiotensin‐aldosterone system.

### 3.2 Quality assessment

Quality assessment was performed using the MINORS score system. The scores of all the included studies ranged from 13 to 16 points, which were acceptable for the present meta‐analysis (Table 2).

**TABLE 2:** Quality assessment of included studies

Study	I	II	III	IV	V	VI	VII	VIII	Total
M Voskuil, 2011	2	2	2	2	2	2	2	0	14
H Ahmed, 2012	2	2	2	2	2	2	1	0	13
W Li, 2013	2	2	2	2	2	1	2	0	13
M Hamza, 2014	2	2	2	2	2	2	1	0	13
J Poss, 2015	2	2	2	2	2	2	1	0	13
S Ewen, 2015	2	2	2	2	2	2	2	0	14
LC Dobrowolski, 2018	2	2	2	2	2	2	2	2	16
L Feyz, 2020	2	2	2	2	2	2	2	0	14
CJ Kim, 2021	2	2	2	2	2	2	2	0	14
F Mahfoud, 2021	2	2	2	2	2	2	2	0	14
C Ott, 2017	2	2	2	2	2	0	2	2	14
M Ezzahti, 2014	2	2	2	2	2	2	1	0	13
D Hering, 2012	2	2	2	2	2	2	1	0	13
D Hering, 2013	2	2	2	2	2	2	2	0	14
D Hering, 2016	2	2	2	2	2	2	2	0	14
J Rosa, 2017	2	2	2	2	2	2	2	2	16
U Kampmann, 2017	2	2	2	2	2	2	1	0	13
C Tsioufis, 2014	2	2	2	2	2	2	2	0	14
OU Bergland, 2020	2	2	2	2	2	2	2	0	14
Y Vuignier, 2018	2	2	2	2	2	2	2	0	14

Numbers I–VIII in heading represented: I, a clearly stated aim; II, inclusion of consecutive patients; III, prospective collection of data; IV, endpoints appropriate to the aim of the study; V, unbiased assessment of the study endpoint; VI, follow‐up period appropriate to the aim of the study; VII, loss of follow up less than 5%; VIII, prospective calculation of the study size.

### 3.3 Effect of RDN on plasma renin and blood pressure

**FIGURE 2:**
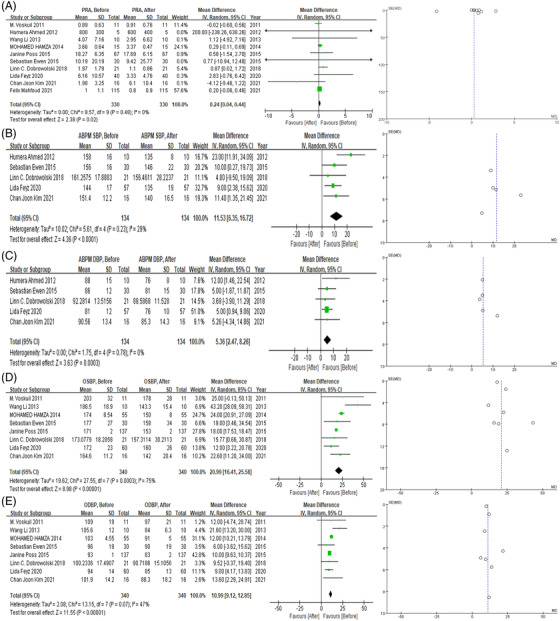
(A) Forest plot and funnel plot of overall PRA level before and after RDN. (B) Forest plot of ambulatory SBP before and after RDN. (C) Forest plot of ambulatory DBP before and after RDN. (D) Forest plot of office SBP before and after RDN. € Forest plot of office DBP before and after RDN. PRA, plasma renin activity; RDN, catheter‐based renal denervation; SBP, systolic blood pressure; DBP, diastolic blood pressure

Comparisons of plasma renin activity levels before and after RDN were extracted in ten studies (n = 330). As presented in Figure 2, pooled PRA showed a significantly decrease after RDN (0.24 ng/mL/h, 95% CI 0.04 to 0.44, *P* = .02). No apparent heterogeneity or publication bias was observed (I^2^ = 0, Egger test = 0.4210, Begg test = 0.7205).

Blood pressure data were subsequently extracted from those ten studies, out of which 5 (n = 134) reported on changes in ambulatory blood pressure, and 8 (n = 340) reported on office blood pressure. As shown in Figure2, both 24‐hour mean systolic blood pressure (SBP) and 24‐h mean diastolic blood pressure (DBP) significantly decreased after RDN (11.53 mmHg, 95% CI 6.35‐16.72, *P* < .0001; 5.36 mmHg, 95% CI 2.47‐8.26, *P* = .0003, respectively). No apparent heterogeneity or publication bias was observed (I^2^ = 29%, Egger test = 0.8672, Begg test = 0.8065; I^2^ = 0, Egger test = 0.5207, Begg test = 0.2207, respectively). Office SBP and DBP also showed reductions after the procedure (20.99 mmHg, 95% CI 16.41‐25.58, *P* < .0001; 10.99 mmHg, 95% CI 9.12 to 12.85, *P* < .0001, respectively). Relatively high heterogeneity was observed in office SBP, and no apparent publication bias was seen (I^2^ = 75%, Egger test = 0.4963, Begg test = 0.5362; I^2^ = 47%, Egger test = 0.5015, Begg test = 0.5362, respectively).

Meta‐regression analyses were performed between baseline PRA and changes in BP values after the surgery. Baseline PRA was positively associated with 24‐hour SBP reduction (coefficient = 0.017, 95% CI 0.002‐0.032, *P* = .025, Table S1).

### 3.4 Effect of RDN on plasma aldosterone

**FIGURE 3:**
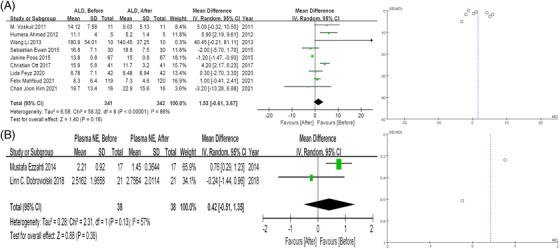
(A) Forest plot and funnel plot of overall PAC level before and after RDN. (B) Forest plot and funnel plot of overall norepinephrine level before and after RDN. PAC, plasma aldosterone concentration; RDN, catheter‐based renal denervation

Changes in plasma aldosterone levels were available in nine studies (n = 341). As displayed in Figure 3A, plasma aldosterone levels tended to decrease after RDN, while the change was not statistically significant (1.53 ng/dL, 95% CI ‐0.61 to 3.67, *P* = .16). High heterogeneity was observed between these studies (I^2^ = 86%). No significant publication bias was found (Egger test = 0.1276, Begg test = 1.0830).

### 3.5 Effect of RDN on plasma catecholamines

Two studies referred to plasma norepinephrine changes after RDN (n = 38). As shown in Figure 3B, no significant difference was found after RDN (0.42 nmol/L, 95% CI ‐0.51 to 1.35, *P* = .38) with moderate heterogeneity (I^2^ = 57%).

### 3.6 Effect of RDN on serum and urinary electrolytes

**FIGURE 4:**
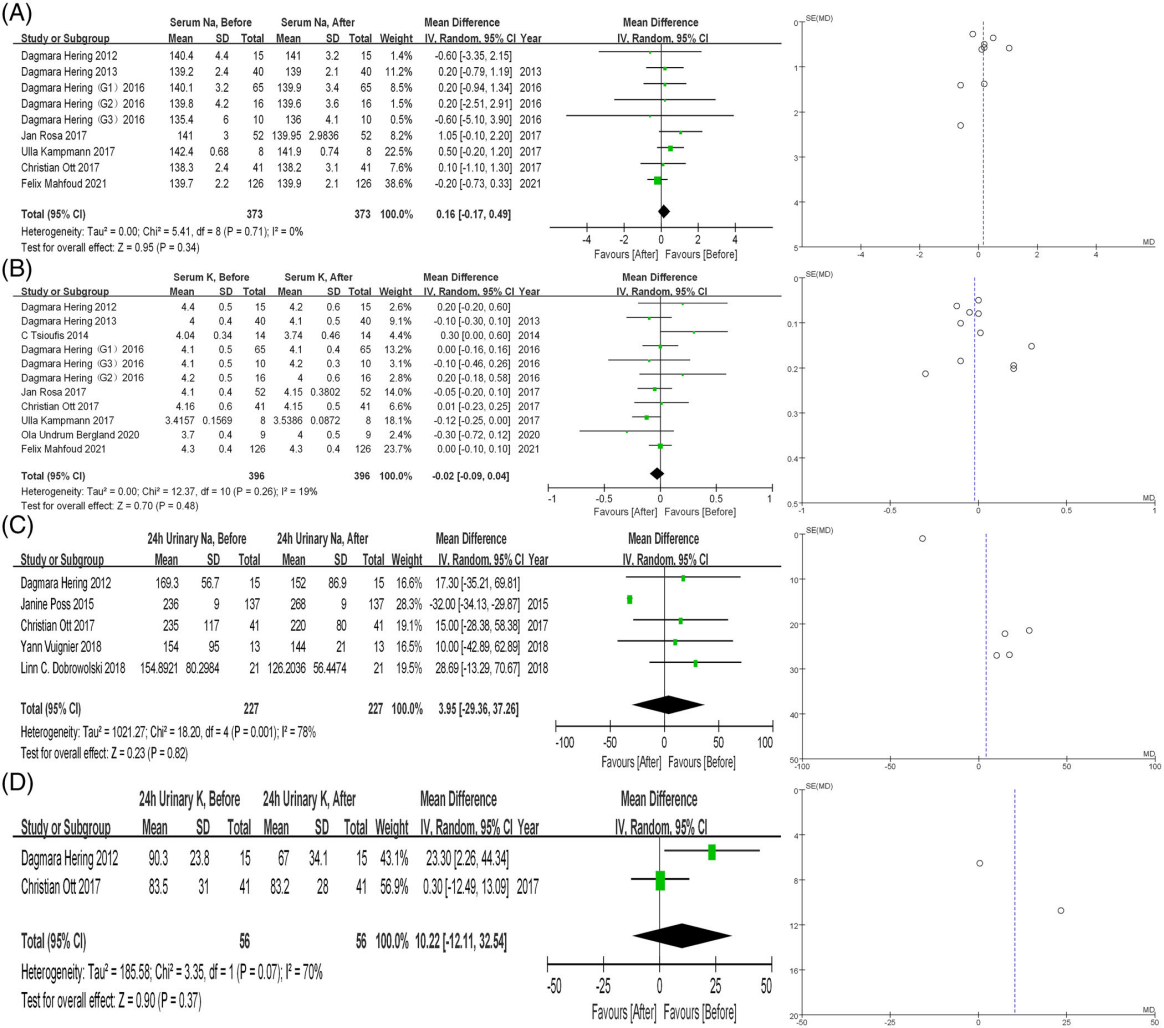
(A) Forest plot and funnel plot of overall serum sodium level before and after RDN. (B) Forest plot and funnel plot of overall serum potassium level before and after RDN C. Forest plot and funnel plot of overall 24 h urinary sodium level before and after RDN D. Forest plot and funnel plot of overall 24 h urinary potassium level before and after RDN. RDN, catheter‐based renal denervation

Seven studies reported changes in serum sodium after RDN (n = 373) and nine studies reported on serum potassium (n = 396), with one study containing three subgroups. Neither serum sodium nor serum potassium was significantly altered (0.16 mmol/L, 95% CI ‐0.17 to 0.49, *P* = .34; ‐0.02 mmol/L, 95% CI ‐0.09 to 0.04, *P* = .48, respectively) after RDN with low heterogeneity (I^2^ = 0; I^2^ = 19%, respectively). The details were presented in Figure 4A and Figure 4B. No significant publication bias was found (Egger test = 0.9702 and Begg test = 1.7485 for sodium; Egger test = 0.3387 and Begg test = 0.7555 for potassium).

Five studies reported data on changes in 24 h urinary sodium after RDN (n = 227). As shown in Figure 4C, no significant change was found (3.95 mmol/24 h, 95% CI ‐29.36 to 37.26, *P* = .82) with high heterogeneity (I^2^ = 78%). However, potential publication bias was detected (Egger test = <0.001, Begg test = 1.5376). Further trim‐and‐fill analysis was conducted to adjust potential publication bias, resulting in estimated MD = ‐9.63 (95% CI ‐33.22 to 13.96, Figure S1). As for 24 h urinary potassium, data was available in two studies (n = 56), and no significant difference was found (10.22 mmol/24 h, 95% CI ‐12.11 to 32.54, *P* = .37) with relatively high heterogeneity (I^2^ = 70%, Figure 4D).

## 4 DISCUSSION

To the best of our knowledge, this study is the first meta‐analysis discussing the effects of catheter‐based RDN on renin, aldosterone, catecholamines, sodium and potassium. Our study indicated that plasma renin significantly decreased after catheter‐based RDN, while no significant change was found regarding plasma aldosterone, catecholamines, serum and urinary sodium and potassium.

Afferent and efferent nerves in the kidney are an important part of the whole nervous system, which are closely associated with the pathophysiology of hypertension.^34^ Afferent sensory nerves are mostly located in the renal pelvic area,^35^ functioning by projecting signals to the central nervous system, thus regulating systemic sympathetic activity, increasing total vascular resistance and raising BP. In contrast, efferent sympathetic nerves distribute widely in all parts of renal vasculature and nephrons, innervating three general targets in the kidney, including juxtaglomerular apparatus, vascular smooth muscle, and the entire tubular system.^36^ Therefore, the activation of efferent nerves can theoretically affect BP levels in multiple ways: 1. Increasing the secretion of circulating renin from the juxtaglomerular apparatus and activating the renin‐angiotensin‐aldosterone system (RAAS); 2. Constricting vessels, increasing regional vascular resistance, and decreasing blood flow in the kidney; 3. Promoting the absorption of sodium and water, and regulating blood volume. Interruption of the above processes has been considered to partially account for the antihypertensive effect of catheter‐based RDN, which may be potentially reflected in the changes in circulating renin, aldosterone, catecholamines, and serum and urinary electrolytes after the surgery. Various studies have reported relative data, while perspectives of whether such changes existed varied from each other.

### 4.1 RDN and RAAS

The effect of RDN on RAAS has been proved in animal models as presented by previous literature.^37^, ^38^ Nevertheless, conclusions drawn from clinical trials remained inconsistent. From the results of our meta‐analysis we can see, the pooled data on PRA showed a significant decrease after RDN procedure. The robustness of the summarizing results was confirmed by low heterogeneity. Additionally, according to the results of meta‐regression analyses, higher baseline PRA seemed to be associated with higher ambulatory SBP reduction after RDN. Guido and associates^39^ revealed that plasma aldosterone and PRA tended to decease after 3 months, and a statistically significant reduction at the 6 months after RDN. While the BP reduction observed in their study preceded by weeks the changes in RAAS hormones, so the conclusion was drawn that the activation of RAAS was not involved in BP control among patients after RDN. On the contrary, SYPRAL HTN‐OFF MED trial^23^ indicated that plasma aldosterone and PRA decreased significantly after RDN, and higher baseline PRA was associated with greater reduction in both office and 24 h BP after RDN procedure. That study was designed as RCT, and patients enrolled were all off antihypertensive medication, which could exclude the confounding impact that medications brought and increased the reliability of results.

According to our study, plasma aldosterone did not change significantly after RDN. While the results presented great heterogeneity, with an I^2^ up to 86%. As we all know, there were many factors affecting the measurement of RAAS hormones. Antihypertensive drugs were one of the most common influencing factors encountered in clinical settings, which could reduce the accuracy of results.^40^ As reported in SYPRAL HTN‐OFF MED trial,^23^ plasma aldosterone was observed to decrease after RDN, when patients were without medications. We presumed that the uneven quality of studies included might contribute to the negative results in our article, and further studies with rigorous designs are warranted to confirm the effect of RDN on RAAS and the role RAAS plays in BP control after RDN procedure.

### 4.2 RDN and catecholamines

As a marker of the total sympathetic tone, pooled plasma norepinephrine presented no significant change after catheter‐based RDN in our study. Nor did other catecholamines hormones as reported by previous publications.^20^, ^21^ The results from SYMPLICITY‐1 trial showed that renal norepinephrine spillover reduced after RDN.^41^ Several possibilities might account for the negative results in plasma samples: 1. The specific inactivation and metabolism mechanisms, and the short half‐life of plasma catecholamines^42^; 2. Inadequate ablation of sympathetic nerves during RDN; 3. Peripheral catecholamines depend not enough on the innervation of afferent and efferent nerves; 4. Lack of high‐quality evidence to detect such alterations in catecholamines. After all, contradicted result was seen in another study,^25^ that plasma norepinephrine did decrease after RDN.

### 4.3 RDN and electrolytes

Neither change in serum nor urinary electrolytes after RDN was found from our pooled data. Several experimental studies indicated that sodium excretion increased in the short‐term after RDN.^43^ In humans, Ott C and associates^24^ also reported a significant decrease in potassium excretion 1 day after RDN, while no change was observed at the 6‐month follow‐up. It was not surprising to find such alterations were eliminated in the long term, since human bodies possess complicated mechanisms for maintaining electrolytes homeostasis.^44^

### 4.4 Limitations

This study had several limitations. First, heterogeneity associated with pooled plasma aldosterone, plasma norepinephrine, and urinary electrolytes might have reduced evidence quality. In addition, studies lacking available data, or presenting data in inappropriate forms were not included in this study. Too few studies reported on urinary catecholamines data to allow for meta‐analysis. More studies with high quality standards and uniform outcome measures were urgently needed to validate the effect of RDN on BP control.

## 5 CONCLUSION

Catheter‐based RDN appeared to have effects on RAAS to some extent. Plasma renin activity significantly decreased after catheter‐based RDN, while no significant change was observed regarding plasma aldosterone, plasma norepinephrine, and serum and urinary electrolytes. Higher baseline PRA seemed to be related to greater BP reduction after RDN, which indicated that patients with high PRA might be more well‐responsive to the treatment. This study might help to confirm the mechanism of RDN regulating BP, and to target the potential patients.

## AUTHOR CONTRIBUTIONS

Study concept and design‐Xiangyu Yang, Zhipeng Zhang, Xiaoping Chen; Literature search and screen‐Xiangyu Yang, Lede Lin; Data extraction‐Xiangyu Yang, Lede Lin; Analysis and interpretation of data‐Xiangyu Yang, Lede Lin; Drafting of the manuscript‐Xiangyu Yang, Lede Lin; Critical revision of the manuscript for important intellectual content‐Zhipeng Zhang, Xiaoping Chen.

## CONFLICT OF INTEREST

The authors declare that they have no conflict of interest.

## Supporting information

Supporting information

Supporting information

Supporting information
