# Supplementary figures and images for: Effects of catheter‐based renal denervation on renin‐aldosterone system, catecholamines, and electrolytes: A systematic review and meta‐analysis
Source: J Clin Hypertens (Greenwich). 2022 Nov 2;24(12):1537–46. doi: 10.1111/jch.14590 (PMC9731592; doi:10.1111/jch.14590)

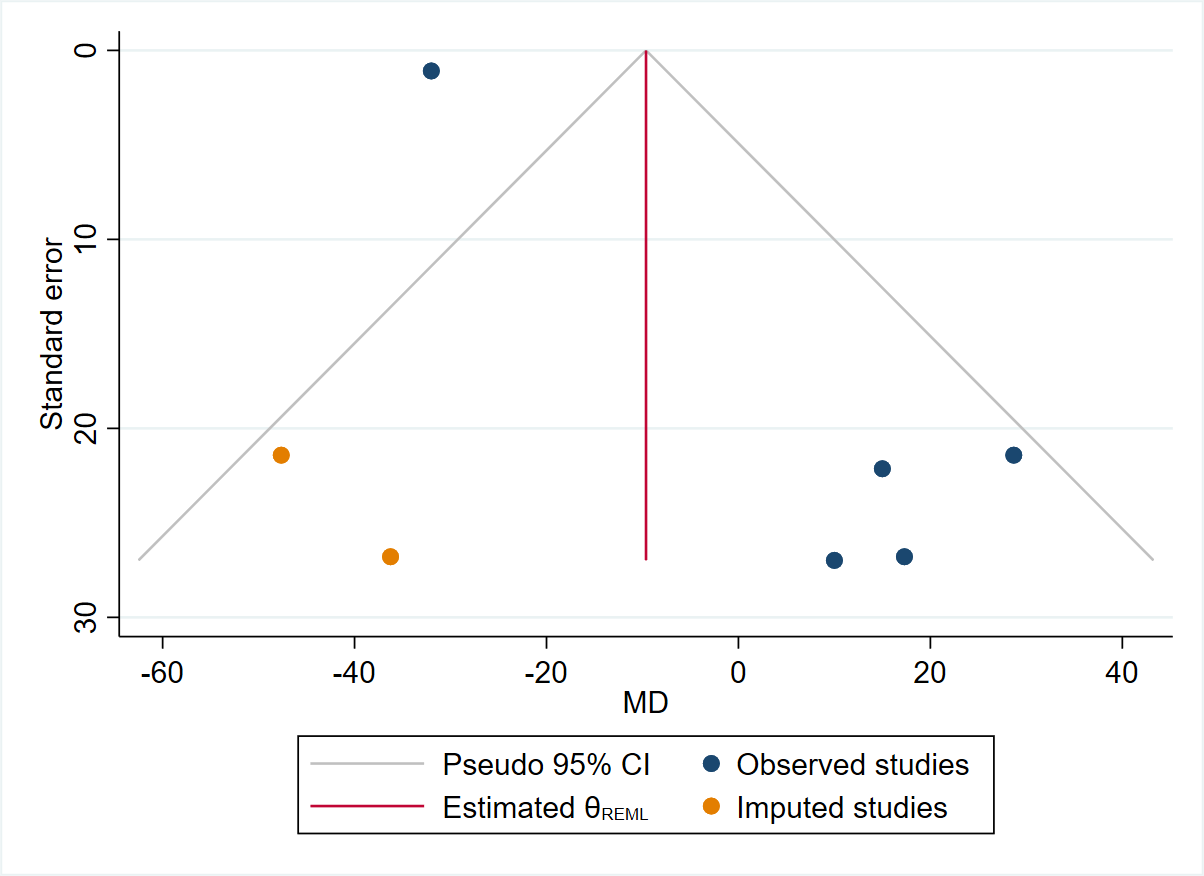

Supplement: Supplementary file 1 — Supporting information [file JCH-24-1537-s001.tif]
