# Supplementary material for: Effects of catheter‐based renal denervation on renin‐aldosterone system, catecholamines, and electrolytes: A systematic review and meta‐analysis
Source: J Clin Hypertens (Greenwich). 2022 Nov 2;24(12):1537–46. doi: 10.1111/jch.14590 (PMC9731592; doi:10.1111/jch.14590)
Supplement: Supplementary file 2 — Supporting information [file JCH-24-1537-s003.docx]

**Table S1. Meta-regression analyses for baseline PRA and BP reductions**

|  | Coefficient | 95% Interval, low | 95% Interval, high | *P* value |
| --- | --- | --- | --- | --- |
| *ABPM SBP* | | | | |
| Baseline PRA | 0.017 | 0.002 | 0.032 | 0.025 |
| *Office SBP* | | | | |
| Baseline PRA | -0.377 | -1.337 | 0.583 | 0.441 |
