# Supplementary material for: Effects of catheter‐based renal denervation on renin‐aldosterone system, catecholamines, and electrolytes: A systematic review and meta‐analysis
Source: J Clin Hypertens (Greenwich). 2022 Nov 2;24(12):1537–46. doi: 10.1111/jch.14590 (PMC9731592; doi:10.1111/jch.14590)
Supplement: Supplementary file 3 — Supporting information [file JCH-24-1537-s002.docx]

**Supplementary file 1: Search strategy for each database**

**Pubmed:**

#1. (renal denervation[MeSH Terms]) OR (kidney denervation[MeSH Terms]) OR (renal sympathetic denervation[MeSH Terms]) OR (renal denervation) OR (kidney denervation) OR (renal sympathetic denervation)

#2. (renin–angiotensin system) OR (renin-angiotensin-aldosterone system) OR (renin) OR (angiotensin) OR (aldosterone)

#3. (Adrenalin) OR (Noradrenalin) OR (Epinephrine) OR (Norepinephrine) OR (Catecholamines) OR (Orciprenaline) OR (Metanephrine)

#4. (sodium) OR (sodium chloride) OR (salt) OR (Na) OR (potassium) OR (K)

#5. #2 OR #3 OR #4

#6. #1 AND #5

**Total:** 2426 results

**Embase:**

#1: 'kidney denervation'/exp OR 'renal sympathetic denervation'/exp OR 'renal denervation'/exp OR 'kidney denervation' OR 'renal sympathetic denervation' OR 'renal denervation'

#2: 'renin–angiotensin system' OR 'renin angiotensin aldosterone system' OR renin OR angiotensin OR aldosterone

#3: adrenalin OR noradrenalin OR epinephrine OR norepinephrine OR catecholamines OR orciprenaline OR metanephrine

#4: sodium OR (sodium AND chloride) OR salt OR na OR potassium OR k

#5: #2 OR #3 OR #4

#6: #1 AND #5

**Total:** 2768 results

**Web of science:**

#1: ALL= (renal denervation OR kidney denervation OR renal sympathetic denervation)

#2: ALL= (renin-angiotensin system OR renin-angiotensin-aldosterone system OR renin OR angiotensin OR aldosterone)

#3: ALL= (adrenalin OR noradrenalin OR epinephrine OR norepinephrine OR catecholamines OR orciprenaline OR metanephrine)

#4: ALL= (sodium OR sodium chloride OR salt OR Na OR potassium OR K)

#5: #2 OR #3 OR #4

#6: #1 AND #5

**Total:** 2352 results
